# From Neoantigens to Nanocarriers: Modern Methods and Modalities in Using Peptides for Cancer Vaccination

**DOI:** 10.1021/acs.biochem.5c00720

**Published:** 2026-04-09

**Authors:** Aleah Harris Treiterer, Blaise Robinson, Sean Huggins, Blaise R. Kimmel

**Affiliations:** † Department of Chemical and Biomolecular Engineering, 142712 The Ohio State University, Columbus, Ohio 43210, United States; ‡ Department of Chemistry, 2647 The Ohio State University, Columbus, Ohio 43210, United States; § Center for Cancer Engineering, Ohio State University Comprehensive Cancer Center, The Ohio State University, Columbus, Ohio 43210, United States; ∥ Pelotonia Institute for Immuno-Oncology, Ohio State University Comprehensive Cancer Center, The Ohio State University, Columbus, Ohio 43210, United States

**Keywords:** cancer, vaccinations, peptides, therapeutic, prophylactic, antigens

## Abstract

The success of cancer vaccination depends on the ability of therapeutics to sustain prolonged immune activation, leading to the destruction of tumor cells. However, only a few therapeutic cancer vaccines have been FDA-approved due to challenges in targeting and eliciting a sufficiently strong immune response. Peptides have emerged as promising drugs owing to their ability to interact with cell-surface receptors and their low manufacturing cost. Despite the peptides’ positive characteristics, additional research is needed to develop more effective methods for using peptides to stimulate the immune system for a sustained period to induce tumor cell regression. This review focuses on recent work in peptide-based vaccine design and development, aiming to determine the optimal formulation of peptide vaccines by identifying and isolating neoantigens for tumor targeting, thereby delivering peptide antigens to specific locations. The expansion of the current landscape of cancer treatments, including peptide vaccines and combination therapies, is revolutionizing the possibilities for patient care and treatment.

## Graphical abstract

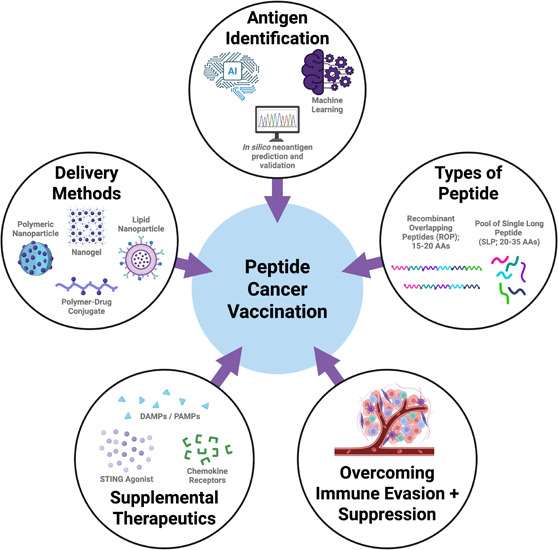

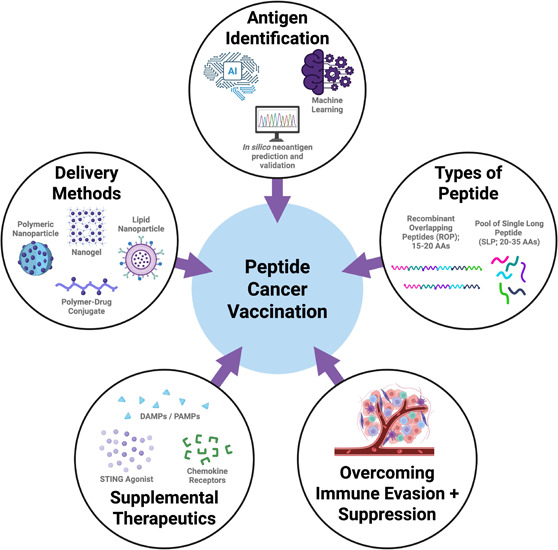

## Introduction

Cancer affects millions of people every year, with the National Cancer Institute estimating that more than 2 million cases of cancer will be identified in the US by 2025.^1^ Over the course of a lifetime, the average person has approximately a 40% risk of developing some form of cancer, with the exact risk depending on a wide range of risk factors.^1^ These statistics demonstrate that cancer will influence most of the human population. Although cancer treatments such as chemotherapy and radiation have been used for more than 50 years, more effective treatments are still needed to improve survival rates, especially for cancers that have metastasized. For example, the five-year survival rate of triple-negative metastatic breast cancer is approximately 40%,^2^ and the survival rate of metastatic pancreatic cancer is as low as 4%.^3^ The statistics demonstrate the large issue of poor prognosis of metastatic cancers, demonstrating the intense need for more effective and targeted methods for treating cancerous tumors.

The most common cancer treatments currently are surgery, chemotherapy, or radiation, with most cancer treatments incorporating a combination of these approaches. Chemotherapy destroys cancer cells as these molecular agents destroy cells that have high rates of proliferation, while radiation treats cancer by delivering high-energy radiation onto cancer cells and tissue.^4^ Targeted treatments, such as monoclonal antibodies, have been developed recently, allowing the drugs to directly inhibit or reverse some cancer functions. These new treatments are more effective due to the ability to provide specificity within targeted cells or proteins, as opposed to destroying any cell with a high rate of division. For example, the monoclonal antibody trastuzumab treats HER2-positive breast cancer by inhibiting the HER2 receptor on the cell surface, thereby preventing cells from dividing and proliferating rapidly. The HER2 receptor is overexpressed in some breast cancer types, particularly HER2-positive cancers. This treatment inhibits one of the primary drivers of cancer cell proliferation, thereby improving prognosis, even in cases of metastasis.

Immunotherapy is a growing field that aims to provide cancer treatment able to both target and destroy cancer cells. One of these techniques is cancer vaccinations, which explore ways to prime the immune system to recognize and destroy cancer cells that would otherwise go unnoticed throughout the body. A key adaptation of cancer is its ability to avoid immune detection in the body. A primary mechanism of cancer-cell recognition is via immune cells, which recognize foreign cells through receptors displayed on the cancer cell surface. Additionally, subsets of these immune cells (such as innate immune cells), can process these surface displayed pieces and display these features as antigens on the surface of the immune cell, which signal to other immune cells which cells should be attacked and eliminated. However, cancer cells evade recognition through strategies such as immune editing and immunosuppression.^5^ Cancer cells have adapted within the tumor environment to evade the immune system, as the cancer cells that successfully evade the immune system are the ones that can proliferate and grow throughout the body. These cells exhibit different immunosuppressive features, such as producing signals that inhibit immune function.^6^ Therefore, the goal of cancer vaccination is to overcome the tumor’s ability to evade detection throughout the body by delivering unique cancer antigens to the immune system, teaching immune cells what features to target for which cells to destroy, while also stimulating the immune system to generate the necessary response that will lead to the destruction of cancer cells.

Multiple materials have been used to develop cancer vaccines, with the main options being mRNA, peptides, and vaccines containing parts or whole cells from cancer patients.^7^ One of the first strategies for investigating cancer vaccination involved isolating patient cancer cells to train the immune system to recognize which cells to attack.^8,9^ Treatment involves isolating the cancer cells from patients, modifying these cells to reduce the ability for these cells to grow and proliferate, and then administering the modified cells back into the patient. These cells are then recognized by immune cells, which initiate an immune response against the cancerous tissue within the body.^8,9^ However, this method of treatment has multiple issues including the vaccine load having low immunogenicity, and difficulty in delivery the engineered tumor cells to APCs.^8,9^ Another treatment option using the patient’s cancer cells involves isolating the patient’s immune cells that can express specific antigens, allowing these cells to express the desired antigens when reintroduced into the body.^10^ While these vaccinations can be effective in eliciting tumor regression, isolating patient cells can be time-consuming and expensive to perform.^11^ Chen et al. explored using cellular vesicles derived from dendritic cells to test more effective and accessible methods, but additional research will be needed to properly optimize treatment with dendritic cells.^12^

An area of growing interest in vaccinations has been in the delivery of nucleic acids to generate an immune response. Nucleic acids are the components that make up RNA and DNA, the genetic material within cells. Within recent years, the focus of these vaccination strategies has shifted to mRNA vaccines, as mRNA is a unique type of RNA that contains the genetic code for producing proteins within cells. During the process of protein synthesis within a cell, the DNA strand is read and transformed into mRNA, which contains the specific codes detailing amino acid sequences for each protein. By delivering mRNA to cells, these cells produce specific proteins of interest or parts of proteins, eliminating the need to directly deliver the protein into the body. The production of the protein of interest then creates an immune response within the body that is specific to the protein encoded by the delivered mRNA. Vaccines made with mRNA, as opposed to DNA, offer a distinct advantage in being transient in nature (i.e., a non-nuclear entry mechanism is required for activation), due to the presence of mRNA within the cellular cytosol instead of being directly integrated into the cell’s gene sequence.^13^ These mRNA vaccines offer distinct advantages including elevated potency, self-adjuvants for functionality in activating the innate immune system, and a relatively safe profile given by the transient nature of mRNA in the body. mRNA can generate proteins within both nondividing and dividing cells.^14^ However, while nucleic acid vaccinations are cheaper to manufacture, they contain multiple challenges in treatment, such as requiring delivery systems that ensure the mRNA is taken up within cells to be used in protein synthesis prior to the mRNA degradation.^15^ A large portion of research within mRNA vaccinations is in delivery systems to find better and more efficient ways to deliver the mRNA to cells.^16–18^

Peptide vaccines share similar goals with mRNA vaccinations – by which these technologies offer the ability to deliver specific proteins or parts of proteins to immune cells, allowing the immune cells to target cancerous tissue within the body. Instead of delivering the code for proteins and relying on cells to synthesize the proteins, peptide vaccinations deliver the parts of proteins directly into the body. Peptides are made up of chains of amino acids and are the components that make up proteins. These peptides are derived from specific proteins or protein fragments expressed by cancer cells. Therefore, the peptides teach immune cells how to target cancer cells within the body. Compared to vaccines utilizing cells isolated from the patient, peptide vaccinations are significantly cheaper and easier to manufacture, increasing the accessibility of the treatment.^5^ Peptide vaccinations also employ the exact mechanism as mRNA vaccinations to generate a targeted immune response, as both involve introducing peptides to immune cells that help the immune system identify and attack cancer cells. Due to the similarity of treatment mechanisms, peptide and mRNA vaccines share similar challenges, as the heterogeneity of tumors can cause the treatments to gradually become less effective. However, peptide vaccinations offer advantages over mRNA vaccines, as the delivery mechanisms used can allow the peptides to circulate throughout the body for longer periods, thereby decreasing the number of treatments patients may need. With mRNA vaccines relying on cells within the body to synthesize peptides, there are clear limitations to methods that ensure the expressed peptides have an extended circulation time. In addition, peptide vaccinations can also be delivered in combination with other molecules or treatments, allowing for dual-drug delivery and potentially increasing the effectiveness of treatment. While mRNA vaccines may include other drugs, the vaccine’s mechanism complicates the delivery of two separate drugs simultaneously (Figure 1).

**Figure 1:**
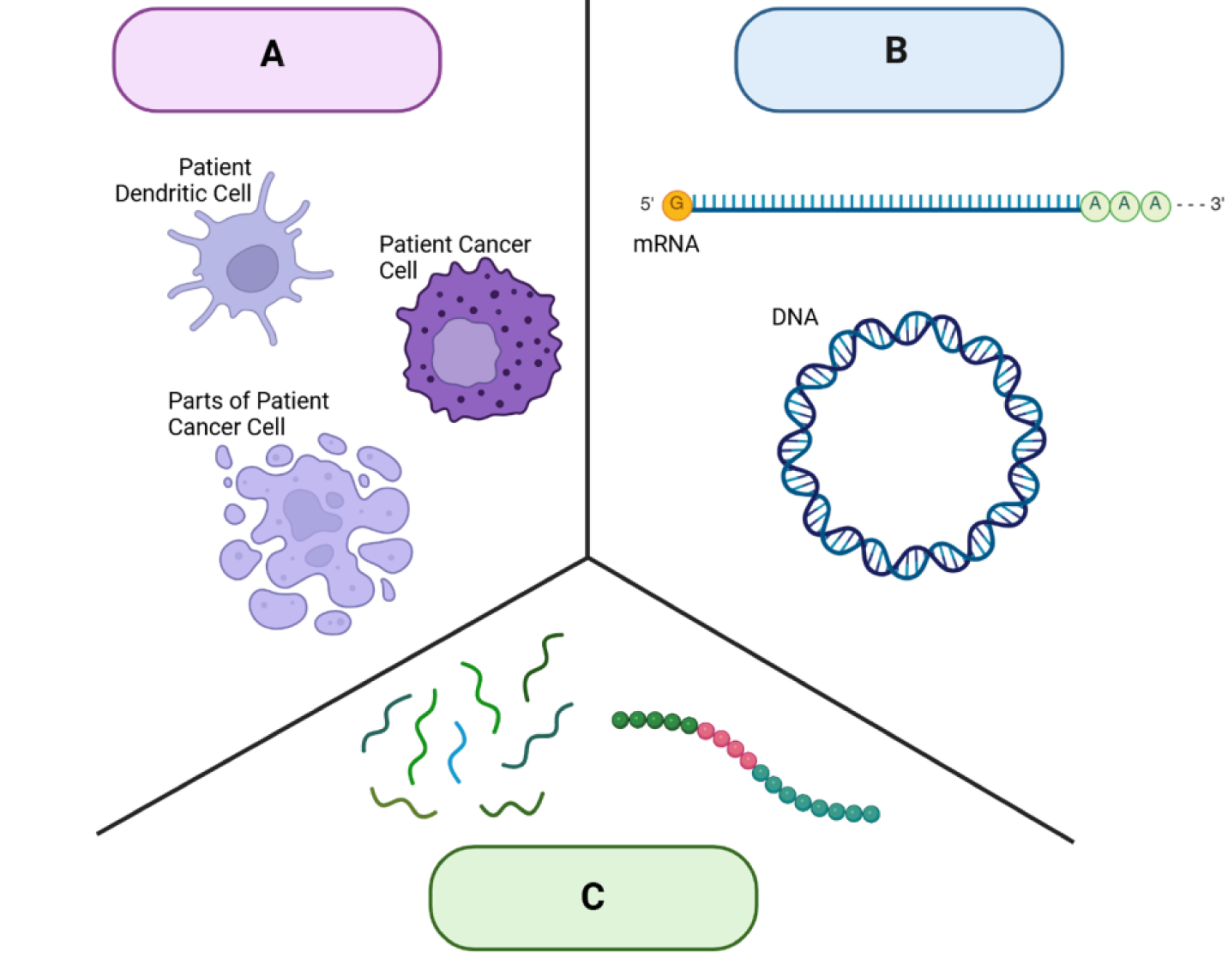
Comparison of different types of cancer vaccinations. (A) Cellular vaccines contain cells or parts of cells isolated from cancer patients, typically either cancerous cells or dendritic cells. These isolated cells are modified and then administered back to the patient to trigger an immune response against cancer cells. While these vaccinations can elicit an immune response that leads to tumor regression, isolating and processing patient cells is time-consuming and expensive. (B) Nucleic acid vaccines are made of either DNA or RNA. The nucleic acid is delivered to cells, allowing the genetic information to be processed and expressed as proteins. Currently, more focus has been placed on mRNA vaccines because the genetic information is not incorporated within the cell’s nucleus; therefore, both dividing and nondividing cells can express the protein of interest. The mRNA vaccines are cheaper and easier to manufacture than cellular vaccines. However, these technologies require a delivery system to ensure the material is successfully transported into the cell. (C) Peptide vaccines contain cancer antigens, designed to stimulate the immune system and induce cancer regression. While these vaccines have high binding affinity for cell receptors and can be combined with other molecules, this approach has known limitations, including difficulty in identifying immunogenic antigens and the potential for peptides to be easily degraded in the body.

The primary focus of this review will be on the recent advances in vaccines constructed with peptides, due to the different advantages peptides have over other vaccine strategies, as detailed in Figure 1.^19^ Working with peptides presents a myriad of disadvantages, such as solubility issues and a short circulation time within the body.^20^ This review will detail the factors involved in engineering a successful peptide vaccination, including the importance of antigen identification and purification, while also exploring different delivery mechanisms and providing examples of the hybridization of cancer therapies.

## Mechanisms of Cancer Vaccination

To create a successful cancer vaccine, the vaccination must elicit a robust T-cell immune response. The primary method of eliciting a strong T-cell response is to have tumor-specific antigens processed and presented by antigen-presenting cells (APCs).^5^ Antigen-presenting cells, or APCs, are cells that process and present foreign particles to instruct immune cells, such as T-cells, on what to identify and destroy within the body.^21^ The complexes within the APCs that present antigens to immune cells are called major histocompatibility complex (MHC) molecules, and these complexes vary depending on the type of immune cell to which the antigen is being presented.^21^ Dendritic cells (DCs) are APCs that act as a link between a nonspecific immune response to an antigen and a specific immune response. DCs internalize the antigen, which is either processed in endosomes to be presented to CD4+ T cells with MHC Class II molecules, or exported into the cytosol and loaded onto an MHC Class I molecule that presents the peptide antigen to CD8+ T-cells (Figure 2). The complex that contains the antigen bound to the MHC molecule is considered the *peptide:MHC* complex.^5^ Should the *peptide:MHC* complex fail to bind together, or if the binding is weak, that will likely decrease the effectiveness of the vaccination. For the immune response to be successful, antigen cross-presentation must also occur, meaning that the antigen is presented to both CD4+ and CD8+ T cells.^22^ If only the presence of CD4+ T-cells is measured after vaccination, the vaccine is likely not to have been successful in generating a meaningful immune response.^22^

**Figure 2:**
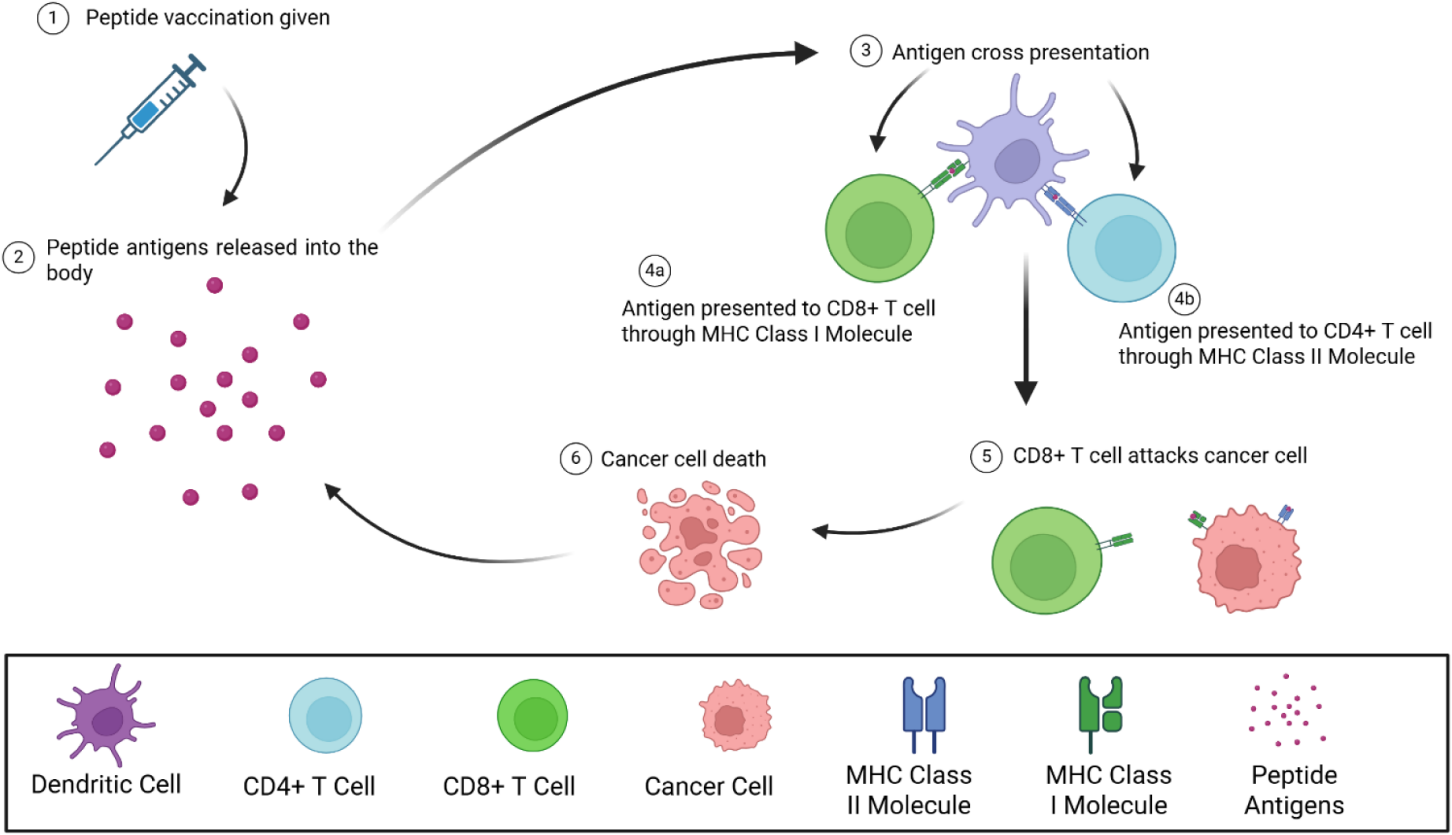
Overview of immune stimulation following the administration of a peptide vaccination. Following the administration of the peptide vaccination, the “drug load” or peptide antigens will be released into the body. The peptides within the body will be taken up by a dendritic cell, and processed either in the cytosol or an endosome, leading to both MHC Class I and Class II presentation. CD8+ and CD4+ T cells can then recognize antigens presented by MHC Class molecules, becoming mature T cells that can attack and destroy cancer cells.

Within cancer vaccinations, there are two main types: prophylactic and therapeutic vaccinations. Prophylactic vaccinations are treatments designed to prevent the formation of cancer within the body.^23^ The only two prophylactic cancer vaccinations that are currently FDA approved protect against vaccines that can cause cancer, where no vaccine has been successful yet at preemptively training the immune system to destroy possible cancer cells.^24^ The development of prophylactic cancer vaccinations could ease the stress on patients, where these technologies may provide preventative cancer treatment, likely easing side effects, and even the need for surgical removal of organs. Therapeutic vaccinations are cancer treatments that train the body’s immune system to recognize and destroy cancerous cells currently present in the body. Currently, there are two prophylactic cancer vaccinations that have FDA approval, and three therapeutic vaccines.^24^ The three FDA-approved therapeutic cancer vaccines are BCG, T-VEC, and sipuleucel-T, each using different strategies to induce an immune response. The BCG vaccine is used to treat bladder cancer and utilizes a drug derived from a bacterium to induce an immune response.^25^ However, this treatment is only effective for treating local cancer, not cancer that has metastasized.^25^ Sipuleucel-T is used to treat prostate cancer patients using immune cells isolated from the patient in a process called leukapheresis to activate the patient’s immune system.^26^ One should note that this process is expensive and time-consuming, which is a current restriction in clinical adoption of these technologies. Research on peptide vaccinations aims to engineer methods for targeting an individual’s immune system without the need to isolate components from the patient. Currently, there is no FDA-approved peptide cancer vaccination; however, Table 1 outlines various clinical trials for peptide vaccinations.

**Table 1:** Clinical Trials of Peptide Cancer vaccinations^43^

Cancer Types	Vaccine Used	Combination	Phase	Clinical Trail Number
Advanced solid tumor (wide range of types)	Personalized neoantigen with sargramostim or GM-CSF SC	Pembrolizumab, cyclophosphamide	I/II	NCT05269381 ^27^
Colorectal and pancreatic cancer	KRAS-targeted long peptide vaccine	Poly-ILCL, nivolumab, ipilimumab	I	NCT04117087 ^28^
Squamous nonsmall cell lung cancer and squamous cell carcinoma of head and neck	Adjusted peptide vaccine (PANDA-VAC)	Pembrolizumab	I	NCT04266730 ^29^
Squamous nonsmall cell lung cancer, squamous cell carcinoma of head and neck, and urothelial bladder cancer	IDO and PD-L1 peptides (IO102-IO103)	Pembrolizumab	II	NCT05077709 ^30^
Stage IIIC-IV melanoma	Personalized neoantigen vaccine	Poly-ICLC	I	NCT05098210 ^31^
Hormone receptor positive HER2 negative breast cancer				
Stage III–IV nonsmall cell lung cancer				
Pancreatic cancer (nonmetastatic resectable pancreatic adenocarcinoma)	Autologous dendritic cells loaded with personalized peptides	Standard of care (SOC) adjuvant chemotherapy, nivolumab	Ib	NCT04627246 ^32^
Ovarian cancer	Multineoepitope vaccine with relevant TAAs (OSE2101)	Pembrolizumab	II	NCT04713514 ^33^
	Neoantigenic peptides	Poly-ICLC, nivolumab	I	NCT04024878 ^34^
Nonsmall cell lung cancer	UCPVax – based on telomerase-derived helper peptides	Nivolumab	II	NCT04263051 ^35^
Melonoma	NeoVax – personalized neoantigen	Poly-ICLC, CDX-301, nivolumab, pembrolizumab	I	NCT04930783 ^36^
Liver cancer	DNAJB1-PKACA Peptide Vaccine	Nivolumab, ipilimumab	I	NCT04248569 ^37^
Glioblastoma	EO2401 peptide vaccine	Nivolumab, bevacizumab	Ib/IIa	NCT04116658 ^38^
Glioma	IDH1R132H peptide vaccine	Avelumab	I	NCT03893903 ^39^
Gastric cancer	OTSGC-A24 peptide vaccine	Nivolumab, Ipilimumab	I	NCT03784040 ^40^
Breast cancer	PVX-410 muli-peptide vaccine	Pembrolizumab, chemotherapy	II	NCT04634747 ^41^
	AE37 peptide vaccine	Pembrolizumab	II	NCT04024800 ^42^

Several factors have hindered FDA approval of cancer vaccinations, including the use of suboptimal adjuvants, tumor heterogeneity that complicates antigen-specific immune responses, loss of tumor antigens, and the inability of certain antigens to elicit sufficiently robust immunity.^5,20^ There are also some challenges associated with the use of peptides for specific drug delivery. Peptides tend to be easily degraded within the body due to the lack of folding stability within the polypeptide backbone, resulting from secondary and tertiary structures, which decreases the circulation half-life of the peptide antigens *in vivo*.^44^ However, peptides also provide unique advantages, such as a distinct ability to act as potent inhibitors of protein–protein interactions, and to trigger intracellular effects with high affinity due to the ability to bind to cell surface receptors.^44^ When considering manufacturing costs, researchers are encouraged to consider the design and use of cost-effective strategies at all stages from idea conception to clinical translation, such as finding new approaches for low-cost peptide production, especially if bioconjugation is required for synthesis. Recent work from the Kimmel lab has collectively highlighted the importance of carefully selecting bioconjugation chemistries, cleavable linkers, and carrier scaffolds – where these design rules offer the ability to enhance the potency and selectivity of immunotherapy prodrugs.^45^ Engineering solutions can be utilized to overcome the challenges of working with peptides, enabling the creation of successful cancer vaccinations that maintain specificity and are cost-effective to produce.

Peptide vaccinations consist of amino acids from either tumor-specific or tumor-associated antigens. Tumor-specific antigens (TSA) are specific to cancer cells and are more difficult to identify. Tumor-associated antigens (TAA) are antigens found in both healthy cells and cancer cells, but typically have elevated levels in cancer cells, such as prostate-specific antigen (PSA) in prostate cancer. Both TSA and TAA antigens are presented to immune cells through processing by APCs, teaching the immune cells what to attack and clear from the body. TAAs are easier to identify and target; however, using TAAs runs the risk of creating off-target effects because healthy cells also contain antigens, so the immune cells might target healthy cells in addition to cancer cells. Section 2 will explore the new methodologies being developed to identify and determine the best antigens for generating robust immune responses.

For a vaccination to be effective, the vaccine must be able to activate both CD8+ T cells and CD4+ T cells through the process of antigen cross-presentation. For example, Ott et al. explored the combination of whole-exome sequencing with RNA sequencing to create specific neoepitope predictions.^36^ These predictions were then used to create peptides that were unique to the patient’s human leukocyte antigen (HLA) type. When these peptides were delivered *ex vivo*, a CD4+ antigen-specific response was observed, but no detectable CD8+ antigen response was noted. However, this could be because the peptides used contained more CD4+ epitopes than CD8+ epitopes.^46^ This study highlights the challenge of developing a vaccination that can activate both CD8+ and CD4+ T cells, which is essential for creating a successful prophylactic or therapeutic cancer treatment. The presence of CD4+ T cells indicates that the antigens were successfully processed within the endosomes of DCs but were unable to be processed in the cytosol of the DCs. Several reasons could have caused the lack of cross-presentation, the most likely being that not enough CD8+ epitopes were used to create the peptides delivered. Therefore, to elicit a strong enough immune response with a cancer vaccination, these designs should include peptides that are processed by endosomes within DCs *and subsequently* within the cytosol. This creates a challenging problem, as the same particle must be processed evenly between two different processes, highlighting one of the primary challenges in the field of cancer vaccination. The ability of the chosen antigens to generate an antigen-specific response indicates that the choice of antigen used in this study is promising.

## All about Antigens

A significant part of this study focuses on the construction of the peptide vaccine itself, but a substantial part of the vaccine’s success is determined by the antigen used. Antigens are broadly defined as any agent that is recognized by the immune system as not belonging to the host tissue.^47^ The agents can be from invading pathogens, such as viruses or bacteria, or can be pathogens developed within the body itself, including those generated by cancer cells.^47^ Suppose an antigen is unable to induce an immune response. In that case, the delivery method or vaccine formulation does not matter, and the vaccine is unlikely to be effective (unless combined with an already proven cancer treatment, such as immune checkpoint inhibitors). As explained previously, there are two main types of antigens: self-antigens (or T**A**As), which are present in both healthy and cancerous cells, but tend to exist at higher levels within tumors, and neo-antigens (or T**S**As), which are only found in cancer cells. The antigens are processed the same way, but the origin of the antigens determines the locations that are targeted by immune cells. Table 2 provides specific examples of antigens, including the cancers from which the antigens are derived.

**Table 2:** Comparing Different TAAs and TSAs^24,70^

	Class/Category of Tumor Antigen	Description	Example Antigens	Example of Cancers Containing Antigen
Tumor-associated antigens (TAA)	Overexpressed antigens (associated with oncogenes)	Antigen is expressed at higher levels in tumor cells than in normal cells	RAGE-1	Pancreatic,^48^ lung,^49^ breast, prostate, colorectal, gastric, liver^49^
			hTERT	Breast, skin, thyroid,^50^ glioblastoma^51^
			HER2	Breast, gastric, gastroesophageal, nonsmall-cell, endometrial, ovarian^52^
			Mesothelin	Mesothelioma, ovarian, pancreatic, lung, breast, cholangiocarcinoma, bile duct carcinoma, gastric cancer^53,54^
	Differentiation antigens	Antigens are expressed in both tumor and normal cells, but only in specific normal cells	Tyrosinase	Melanoma,^55^ neuroblastoma^56^
			gp100	Melanoma^57^
			MART-1	Melanoma^58^
			Prostate-specific antigen (PSA)	Prostate^59^
Tumor-specific antigens (TSA)	Oncogenic Viral Antigens	Abnormal expression of antigen due to viral infection	EBV LMP-1	Nasopharyngeal carcinoma, gastric, lymphoma^60^
			HPV-E6/E7	Cervical,^61^ head neck squamous cell carcinoma, anal, penile, vaginal, vulvar^62^
			HTLV-1	Adult T-cell leukemia/lymphoma (ATL),^63^ endometrial^64^
	Tumor-specific antigens	Antigens expressed due to mutations within tumor cells	KRAS	Nonsmall cell lung, colorectal, pancreatic^65^
			NRAS	Melanoma, lung adenocarcinoma, colon, pancreatic, leukemia^66^
			ETV6	Leukemia,^67^ lymphoma^68^
			NPM/ALK	Lymphoma, lung^69^

While self-antigens may be easier to identify and isolate, neo-antigens have the advantage of limiting off-target effects due to the unique association of self-antigens with cancer cells. Some neo-antigens are common across cancer types or are present in different patients with the same cancer type, allowing for the development of exact strategies to target these cells of interest. While others, such as UBR4 and PRKDC, are neoantigens that have only been identified in a specific cancer type.^71^ There has been an increasing interest in identifying and isolating neo-antigens unique to patients and all types of cancers. While “universal” neoantigens are beneficial for a select number of patients with cancers that express these antigens, many cancers remain untreatable with universal antigens, because the neoantigen landscape for these cancers is dominated by rare or patient-specific mutations. The ability to tailor peptide vaccinations to a specific patient’s cancerous mutations could aid in the treatment of both rare and more common forms of cancer.

Certain mutations within oncogenes are shared across multiple patients due to similar mutations within specific types of cancer. These neoantigens are classified as “public” neoantigens.^72^ While there has been a primary focus in recent years on personalized neo-antigens detected from an individual’s tumor, we note the importance of evaluating the impact that public neo-antigens have on yielding a robust immune profile for patients. Public neo-antigens are commonly present in driver oncogenes that facilitate either tumorigenesis (the creation and spread of cancer cells) or drive continued tumor growth, with a notable example being present in the TP53 family. The *TP53* gene is involved in regulating the cell cycle, including cell apoptosis, when detecting abnormalities or damage.^73^ Should the gene become mutated, one of the mechanisms that prevents uncontrolled cell growth has been interrupted, allowing the mutated gene to be present in many different types of cancer.^74^ Additionally, public neoantigens with shared MHC restrictions often contain overlapping MHC-I and MHC-II epitopes, which are crucial for inducing CD4+ and CD8+ T cell responses. The usefulness of different public neo-antigens is determined by the frequency of the mutation and the frequency of expression in an HLA allele within each tumor population.^72^ In addition, with public neo-antigens being more likely to be clonally expressed, this decreases the likelihood of antigen-loss tumor escape variants, which are cancer cell variants that no longer express the antigen being targeted by treatment – implying that treatment against recognized cancer cells may become more effective over time.

In recent years, significant advances have been made in identifying and isolating neo-antigens unique to various forms of cancer. Additionally, studies have been conducted to determine whether identified antigens can stimulate the immune system to destroy cancerous tissue. However, a main challenge within neoantigen prediction is identifying peptide sequences that are able to bind and be presented by HLA molecules.^75^ A common historic approach for neo-antigen prediction is *in silico* sequencing. However, this method of neoantigen prediction has been unable to produce significant numbers of antigens that perform successfully within a clinical setting. The discrepancy within immunogenicity of neoantigens stems from various different reasons including inefficient antigen process, weak recognition by T cells, an immunosuppressive tumor environment, and antigens without enough differences to register as “nonself” from other immune cells.^76^ Mass spectrometry is another strategy for identifying neo-antigens. Liquid chromatography-tandem mass spectrometry (LC-MS/MS) has been used to determine the most immunogenic antigens of tumor cells.^77^ Figure 3 provides a comparison of the main historical methods used to determine possible neo-antigen sequences. The process works by evaluating the ligandome of cells, which is the range of peptides expressed by specific MHC molecules from a particular cell.^72,78^ By being able to sort and identify peptides expressed by MHC molecules, researchers can determine which potential peptides will be presented to T cells, thereby generating immune responses.^79^

**Figure 3:**
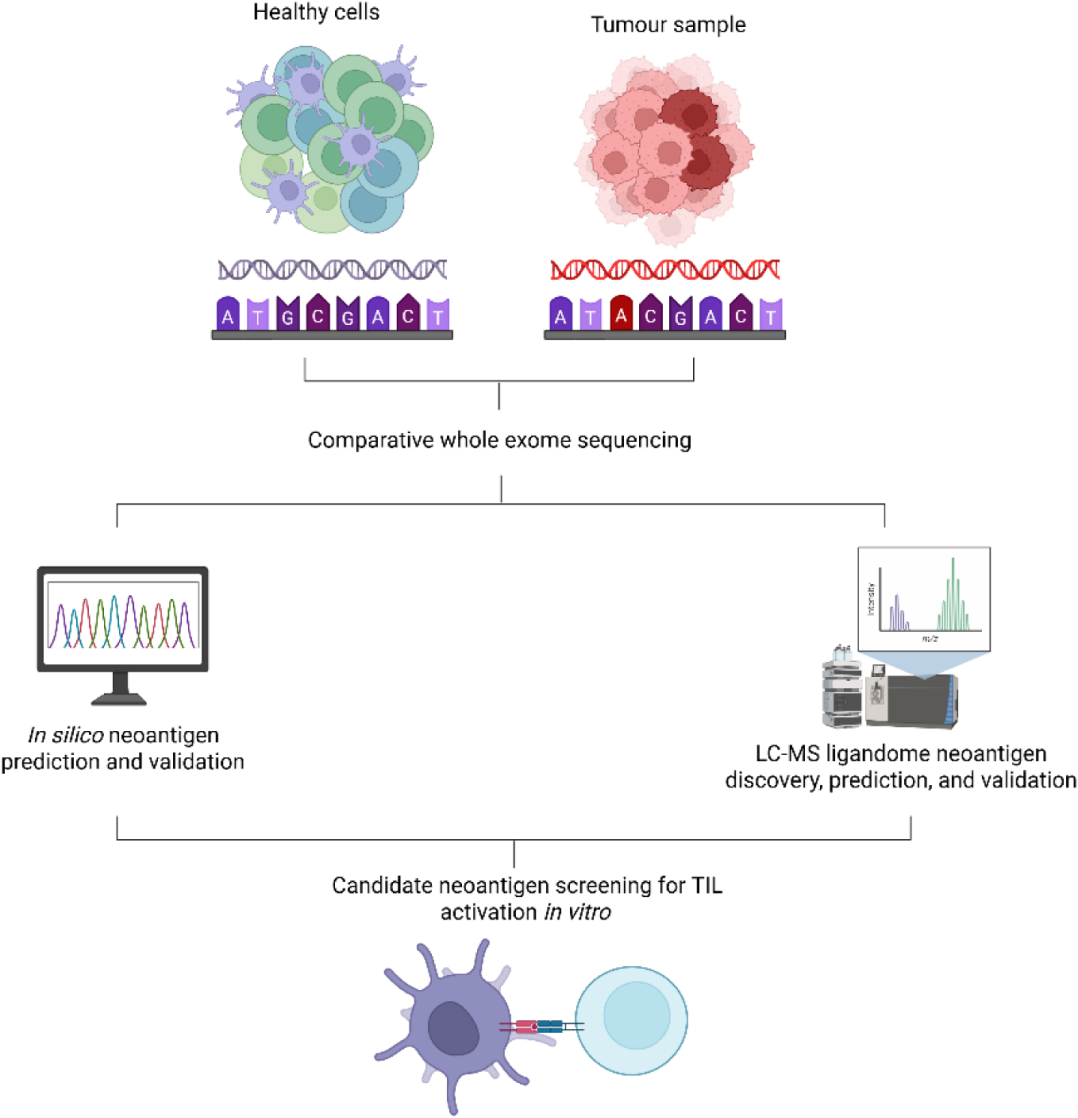
Comparing *in silico* vs LC-MS neo-antigen screening methods. Both *in silico* and LC-MS neoantigen screening methods compare healthy cells to tumor samples to identify differences in the two genetic sequences. *In silico* analysis methods typically involve computer-based screening, whereas LC-MS methods involve eluting MHC peptide ligands prior to LC-MS to identify potential neoantigen candidates. Following the identification of potential neo-antigens, the candidates are screened *in vitro* to assess TIL activation.

Despite issues in being able to identify immunogenic peptides using LC-MS, researchers are still reliant on the method as the only way to directly profile HLA-presented peptides.^77^ Manakongtreecheep et al. postulates that one of the issues within the MS acquisition strategy is due to the reliance on data-dependent acquisition (DDA).^77^ The over-reliance on DDA causes MS to have a bias to choose more abundant peptides, possibly causing immunogenic peptides to be missed. To combat the sample-bias in addition to other challenges within MS accuracy, Manakongtreecheep et al. created Pepyrus, a method which uses both DIA and machine learning to construct large peptide libraries that have the ability to include previously undetected neoantigens.^77^ The addition of machine learning within the workflow greatly advanced the predicting and sorting ability that was used to create Pepyrus. As machine learning and AI techniques have improved, the focus within the neo-antigen discovery field has shifted into including the computational field to predict immunogenic antigens.

To combat the challenges of neo-antigen experimental validation, which is both costly and at times inaccurate, researchers have worked on AI and machine learning tools to identify and predict immunogenic neo-antigens. One specific database that has been created to combat the challenges within computational and experimental neo-antigen predictions is TumorAgDB2.0, which integrates neoantigen data, and incorporates the data within the NeoTImmuML prediction tool.^80^ The prediction tool was built first by computing the physiochemical feature of each individual peptide, which was then fed into machine learning algorithms that analyzed the immunogenicity of each peptide sequence based on 78 different features.^80^ The algorithm created by Shao et al. is one of many new computation tools that have been created to screen for immunogenic neo-antigens. Table 3 provides a list of many other machine learning and AI computational tools that have been created to improve on the neo-antigen predictions for cancer vaccinations.

**Table 3:** List of Computational Neo-Antigen Tools

Computational Tool	Purpose	Link to Tool
timsTOF Prosit^81^	Trained to identify and predict immunopeptides and HLA-I peptides using PSM rescoring of MaxQuant results.	https://koina.proteomicsdb.org/
AlphaPept^82^	Python based database to process large high resolution MS data sets, including features such as peptide identification and protein quantification. One drawback is the database only has functionality for DDA proteomics.	https://alphapept.org/
RPEMHC^83^	Deep learning method to predict the binding affinity between peptides and MHC Class I and II molecules based on residue–residue pair encoding.	https://github.com/lennylv/RPEMHC
SNAF^84^	Computational tool to predict possible T cell and B cell antigens by identifying and interpreting classes of splicing neo-antigens.	N/A
TIMS^2^Rescore^85^	Tool that works with timsTOF model to assist with analyzing a sample’s proteome, including immunopeptidomics.	https://github.com/compomics/tims2rescore
UniPMT^86^	Computational tool that predicts binding of the peptide-MHC-TCR complex, the peptide-MHC complex, and the peptide-TCR complex.	N/A
VirusImmu^87^	Machine learning tool to predict B cell epitope immunogenicity. However, it is important to note that this tool is used for virus epitope predictions.	https://github.com/zhangjbig/VirusImmu
CNNeoPP^88^	Prediction model for neoantigen immunogenic classification.	https://github.com/AaronChen007/neoantigen
NeoPrecis-Immuno^89^	Neo-antigen immunogenicity prediction model, specifically shown to assist with predicting patient outcomes within Immune Checkpoint Inhibition treatment.	N/A

Despite the large advances in neo-antigen prediction, there are still low experimental validation of computationally predicted antigens, and not enough understanding of the mechanisms behind peptide immunogenicity.^76^ High-throughput display platforms – particularly within yeast synthetic biology systems – offer complementary routes to screen antigen-binding interactions and immunotherapy candidates functionally, as reviewed in detail by Slaton et al.^90^ These sequence-level design rules are increasingly being coupled to modular discovery platforms, such as innovative yeast display workflows that iteratively evolve immunotherapy candidates with optimized binding and signaling properties. Recent work has shown that the degree of biochemical diversity of amino acids correlates with immunogenicity levels. Calis et al. demonstrated that amino acids with mutations that led to the incorporation of amino acids with large aromatic side chains were more frequently able to trigger an immune response.^91^ Capietto et al. showed that modifications to nonanchor amino acids enhanced *peptide:MHC* stability, also prolonging immunogenicity by increasing the duration of TCR contact.^92^ Additional research has also been conducted on anchor residue mutations, which have shown that the TCR interface is equivalent to neo-antigenic and wild-type peptide sequences, indicating that other characteristics need to be investigated to determine what determines immunogenicity. Additional research is necessary to bridge the gap between computational predictions, and experimental validation to determine the specific characteristics that determine the immunogenicity of different peptides and neo-antigens.

## Types and Deliveries of Peptide Vaccinations

When formulating cancer vaccinations, aside from the specific antigen used in the vaccination, there are two main focuses on the design of the drug itself: the drug load, or what will be delivered, such as the specific peptide that will be used to generate an immune response, and the delivery mechanism. These two focuses can also be hybridized depending on the drug being delivered, as certain characteristics can be utilized to create more efficient delivery. When examining the peptide itself, peptide length is a determinant of immune response success. If a peptide is too short, the peptide fragment can bind to the multihistological complex (MHC) of APCs that lack the secondary signaling ability to complete T-cell activation. Short peptides also tend to be HLA-type restricted and are eliminated faster from the body due to enzymatic digestion. This is important to note that there was no “distinct length” detailed in the literature defining the length of a short peptide, but some researchers dictate that a short peptide contains fewer than 20 amino acids.^93^ Longer peptides allow for broader populations of HLA types and the presence of multiepitope peptides. Similar to the difficulty of defining a short peptide, there has not been a definitive length defined in the literature. However, multiple studies that generated an immune response used lengths longer than 9–11 amino acids.^94^

The HLA molecules play a crucial role in helping the immune system identify what is considered foreign to the body. The greater the diversity of peptides within the vaccine, the higher the likelihood that the vaccine will be able to target all cancer cells, despite the wide range of different antigens expressed by cancers within the body. In turn, this increases the likelihood of antigen cross-presentation, leading to the activation of CD4+ and CD8+ T cells. Additionally, several types of peptides have been investigated to determine which ones elicit the strongest immune response. The first involves selecting peptide sequences from TSAs or TAAs using T-cell epitopes as a template. By using multiple strands of peptides, the vaccine can overcome the issues of tumor heterogeneity and tumor antigen downregulation. Synthetic long peptides (SLPs) are an example of the incorporation of long peptides into cancer vaccinations. The SLP technology uses peptides containing 25–35 amino acid residues and has been shown to elicit a stronger immune response compared to treatment with only the individual antigens from which the SLPs were derived. Ott et al. administered SLP vaccines with candidate neoantigens, adjuvanted by the coadministration of a T-cell receptor agonist, to stimulate the immune system.^46^ The results showed that 67% of patients experienced no disease progression over 25 months, and all patients evaluated showed CD4+ and CD8+ responses that persisted over time.^46^

Although this SLP vaccine was successful in generating immune responses, the design focused on using an agonist with a neoantigen of interest, meaning the vaccine could not stimulate the immune system solely with the delivery of the neoantigen alone.^46^ We note with this design that long peptides cannot directly bind to MHC-I on DCs or non-APCs. Peptides must be processed within DCs before being presented to T-cells. Despite the additional steps required to process vaccines containing SLPs, these vaccines have been shown to generate successful immune responses. For example, a peptide cancer vaccine targeting survivin, an antiapoptotic protein that inhibits apoptosis and is typically upregulated in cancers, was shown to induce CD4+ and CD8+ T cell responses, in addition to stimulating autologous dendritic cells. The vaccine consisted of three different SLPs, each containing eight CD8+ and six CD4+ T cell epitopes, and demonstrated a statistically significant level of tumor eradication *in vivo*.^95^ Another formulation of peptide vaccines utilizes recombinant overlapping peptides (ROPs), which have shown promising preclinical success (Figure 4).

**Figure 4:**
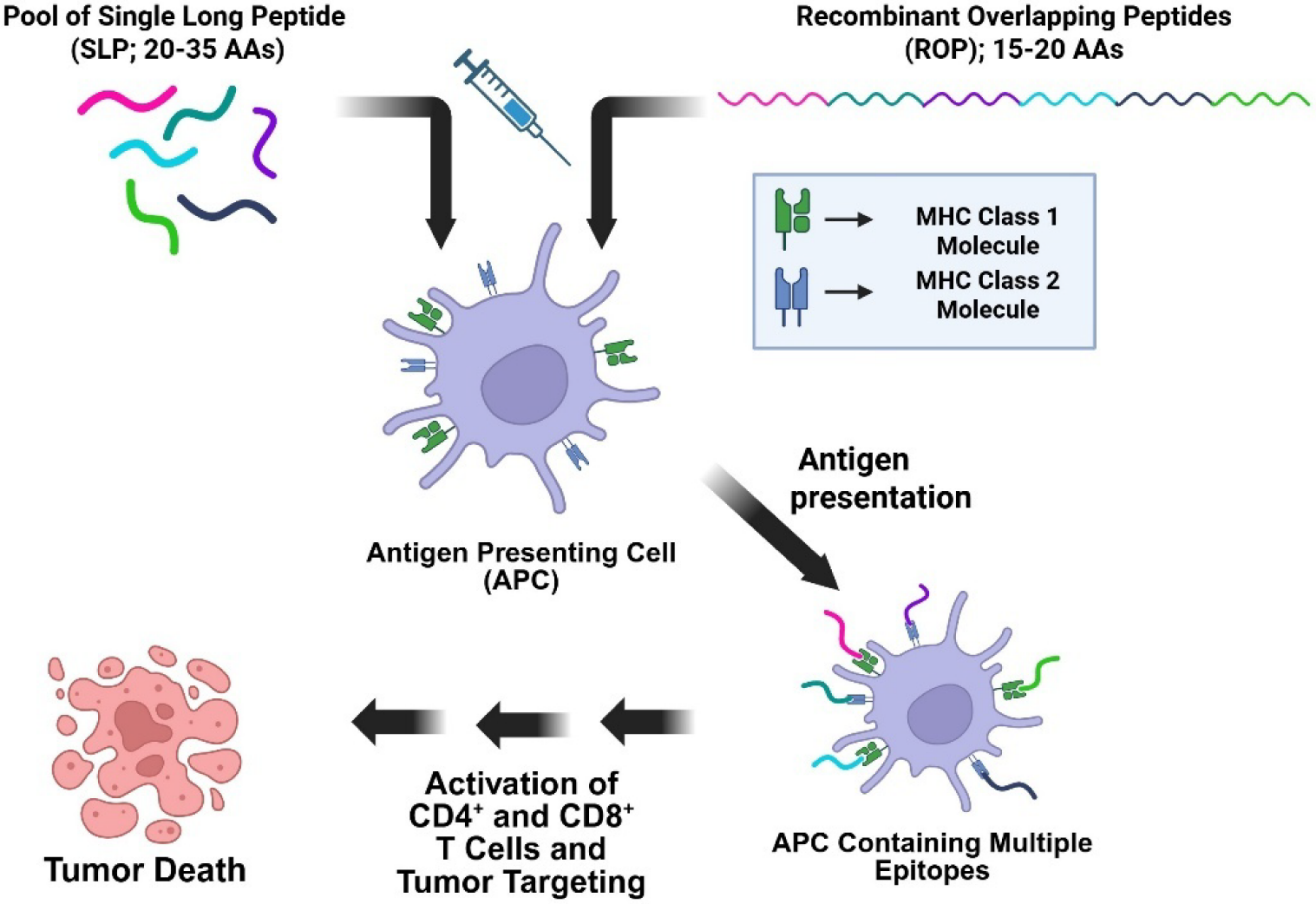
How SLP and ROP vaccinations stimulate the immune system. Both SLP and ROP vaccinations stimulate the immune system by being processed by APCs, leading to the activation of CD4+ and CD8+ T cells. However, SLP vaccinations comprise a pool of peptides, each representing a different antigen epitope, whereas ROP vaccinations contain a single peptide with multiple epitopes encoded within the peptide.

The overlapping sequences of peptides within ROPs are connected by the protease Cathepsin S.^97^ Proteases are enzymes that break apart proteins and peptides, with Cathepsin S being predominantly present in immune cells, such as macrophages and APC.^97^ Within DCs, Cathepsin S degrades a chain that restricts the MHC-II molecule, allowing the MHC-II to create complexes with antigens.^97^ The overlapping region within the vaccine creates diversity in the epitope, especially with MHC-II molecules, and the peptides have been shown to produce a strong immunological response that can also break self-tolerance.^5^ Zhang et al. explored the effectiveness of overlapping peptides in activating both CD8+ and CD4+ T cells and found that overlapping ROP peptides were more successful in activating T cells compared to the whole protein.^98^ However, this study was focused on the treatment of HIV, which differs from cancer cells despite both diseases creating immunosuppressive effects.

Building on prior studies, Cai et al. investigated the effectiveness of ROPs in generating immune responses against melanoma.^96^ In the study, Cai created a peptide strand with ROPs linked using Cathepsin, with the ROPs designed for four different antigens: ovalbumin, a tuberculosis protein, an HPV protein, and survivin.^96^ Vaccines containing ROPs were able to generate an immune response in mice that were given melanoma cells expressing either survivin or the HPV protein, thereby preventing the mice from infection.^96^ While this study demonstrated that vaccination generated an immune response, the authors did not determine the duration of the immune response or whether the dosage would be sufficient to treat an already present tumor. Wang et al. furthered the investigation of the effectiveness of recombinant vaccines, while also tackling the challenge of weak immune responses from peptide vaccinations by incorporating specific LMP2A antigen epitopes, in addition to TLR4 agonist and human IgG1 epitopes within the cancer vaccination.^99^ The researchers utilized in silico methods to determine different antigen epitopes for an EBV antigen, and nasopharyngeal cancer can contain EBV proteins within their cells.^99^ Within both in vitro and in vivo studies, Wang et al. was able to demonstrate successful cellular immune responses and slowed tumor growth within mice following the administration of the recombinant vaccination.^99^ This method could be an interesting avenue to explore for prophylactic vaccines, particularly in situations where there is a high likelihood of developing a specific type of cancer. We note here that ROP vaccinations are produced as a single-chain polypeptide with multiple epitopes. While that formulation is advantageous for manufacturing and FDA approval, these strategies for building vaccine technologies can produce issues with solubility.^5^ In addition, despite some promising results from the studies detailed above regarding the ability of ROPs to stimulate the immune system, additional studies are needed to explore the efficacy of the vaccination strategy within humans. Currently, there is a clinical trial (NCT05104515) ongoing that is exploring the safety and efficacy of the ROP vaccination OVM-200 within humans.^100^ Many different materials and delivery systems have been used to deliver the vaccination payload into the body. For the purposes of this review, the focus is on polymeric, lipid-based, and novel formulations being explored for efficient drug delivery, which are summarized below in Figure 5.^101–145^

**Figure 5:**
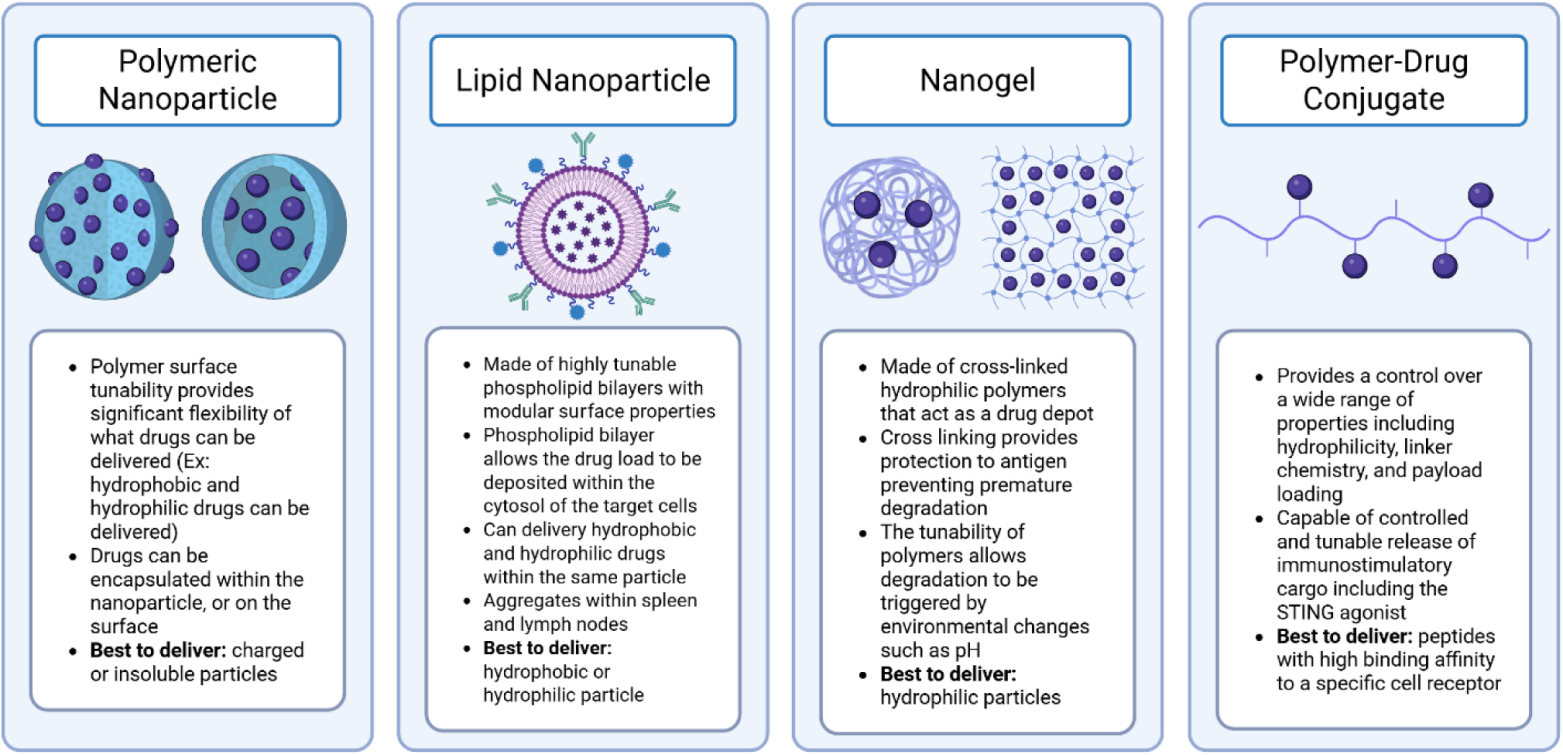
Different vaccine delivery methods for peptide vaccinations. There have been significant advances in drug delivery technologies, and four of the main methods are detailed within the figure. Each specific method has its advantages and drawbacks, which are detailed in the bullet points below each icon.

The method of delivery for vaccinations can be used to mitigate problems such as off-target effects, short circulation times, and drug toxicity of drugs.^5^ For peptide vaccinations, the delivery mechanisms must overcome the initial issues associated with the aforementioned peptide vaccination methods, such as ease of degradation and short circulation times within the body. For example, a delivery mechanism could be engineered to protect peptides from degradation until the peptide cargo reaches the intended target or to prolong the time during which the peptide can circulate throughout the body. Not only would this help solve one of the primary issues with peptide vaccination methodologies, but this could also increase patient compliance, as an increase in circulation time means a reduced number of infusions a patient will need during treatment. The goal of engineering drug delivery mechanisms is to leverage the positive aspects of peptides, such as the engineered high affinity for binding to cell surface receptors, while mitigating or addressing the issues associated with peptides as potential therapeutics and immune antigens. Table 4 details the specific applications of the delivery methods described within peptide vaccinations.

**Table 4:** Examples of Peptide Cancer Vaccinations Administered with Different Delivery Mechanisms

Type of Delivery Method	Application
Polymeric NP^108^	Vaccine formulation: Poly(lactic-*co*-glycolic) acid (PLGA) and dimethyl-dioctadecyl-ammonium bromide (DDAB) nanoparticle
	Drug load: Model antigen (OVA) conjugated on the surface
	Immune Response: Generated CD4+ and CD8+ T cell response in addition to having successful nanoparticle delivery to the lymph nodes^108^
	Additional Information: Did not perform in vivo tumor studies, therefore further research is needed to determine the ability of the nanoparticle to slow cancer progression
Lipid nanoparticles^109^	Vaccine formulation: Cationic liposomes
	Drug load: OVA24 or OVA17 SLPs
	Immune Response: Vaccines were able to both activate T-cells *and* induce an immune response *in vivo* leading to tumor regression
	Additional Information: Cationic liposome vaccination was also compared to PLGA vaccination with same drug load, and the liposome was found to generate a more effective immune response^109^
Nanogels^101,102^	Vaccine formulation: Nanogel made of cationic dextran
	Drug load: SLPs that included cytotoxic C lymphocytes (CTL) and CD4+ T helpers
	Immune Response: Delivery *in vivo* lead to T cell activation^101^
	Additional Information: Preliminary study only, so further investigation within tumor models is needed to determine efficacy of treatment
	Vaccine Formulation: Nanogel made of monomer N-[(2,2-dimethyl-1,3-dioxolane)methyl]acrylamide (DMDOMA)
	Drug load: N/A, this was a study to only investigate delivery mechanism of nanogel
	Additional Information: Shown to completely degrade from hydrolysis within acidic environments, which was hypothesized to provide controlled drug release of anticancer drugs within acidic environments^102^
Polymer-drug conjugates^107,110–113^	Vaccine Formulation: Hydrophilic polymers with cleavable linkers
	Drug Load: Immunostimulatory payloads including targeted STING agonists
	Additional Information: Programmable pH responsive nanocarriers provided a tunable carrier architecture that can be combined with antigen delivery to reprogram the vascular-immune interface and substantially broaden responses to checkpoint inhibitors and adoptive cell therapies – self-assembling nanoparticles with peptide-TLR-7/8a conjugates was engineered to be able to self-assemble no matter the charge of peptide antigen used by conjugating a charge-modifying group and hydrophobic block to either end of the peptide of interest^113^-*in vivo* vaccination was shown to have uptake within APCs and elicit a significant T cell response
	Vaccine Formulation: Self-assembling nanoparticles with peptide-TLR-7/8a conjugates
	Drug Load: Variable peptide antigen
	Additional Information: Nanoparticles were engineered to self-assemble no matter the charge of peptide antigen. The *in vivo* vaccination was shown to have uptake within APCs and elicit a significant T cell response.

### Current Advancements in Peptide Vaccinations

A significant issue with peptide vaccines operating alone is the difficulty in inducing a sufficiently robust immune response to eradicate all tumor cells. Therefore, additional molecules and strategies are needed to work in tandem with peptide vaccines to enhance treatment effectiveness. One strategy is to utilize immune stimulants or adjuvants to enhance the immune system’s response. Typically, this strategy involves conjugating adjuvants with antigens within the vaccine. The most common method is the use of molecular stress signals produced by immune cells, such as pathogen-associated molecular patterns (PAMPs) or damage-associated molecular patterns (DAMPs).^114^ These molecules bind to Pattern Recognition Receptors (PRRs) on the surface of DCs, such as toll-like receptors (TLRs), C-type lectin receptors (CLRs), and NOD-like receptors (NLRs).^115^ PRR activation leads to DC maturation and upregulation of MHC-II expression. This, in turn, creates a costimulatory signal – a signal that can activate separate items in parallel – that releases pro-inflammatory cytokines to bolster the immune response (Figure 6).^119^ In addition to TLR-targeting PAMPs, cytosolic pattern-recognition receptors (e.g., STING agonists and Retinoic Acid-Inducible Gene I Agonists) have emerged as powerful adjuvant targets. Using these technologies (i.e., nanoparticle-based agonist formulations), Wilson et al. have demonstrated the ability of these technologies to normalize the tumor vasculature, enhance T-cell infiltration, and synergize with checkpoint blockade – validating the rational design principles used to engineer carriers that turn innate sensing pathways into potent adjuvants for peptide vaccines.^116–118^

**Figure 6:**
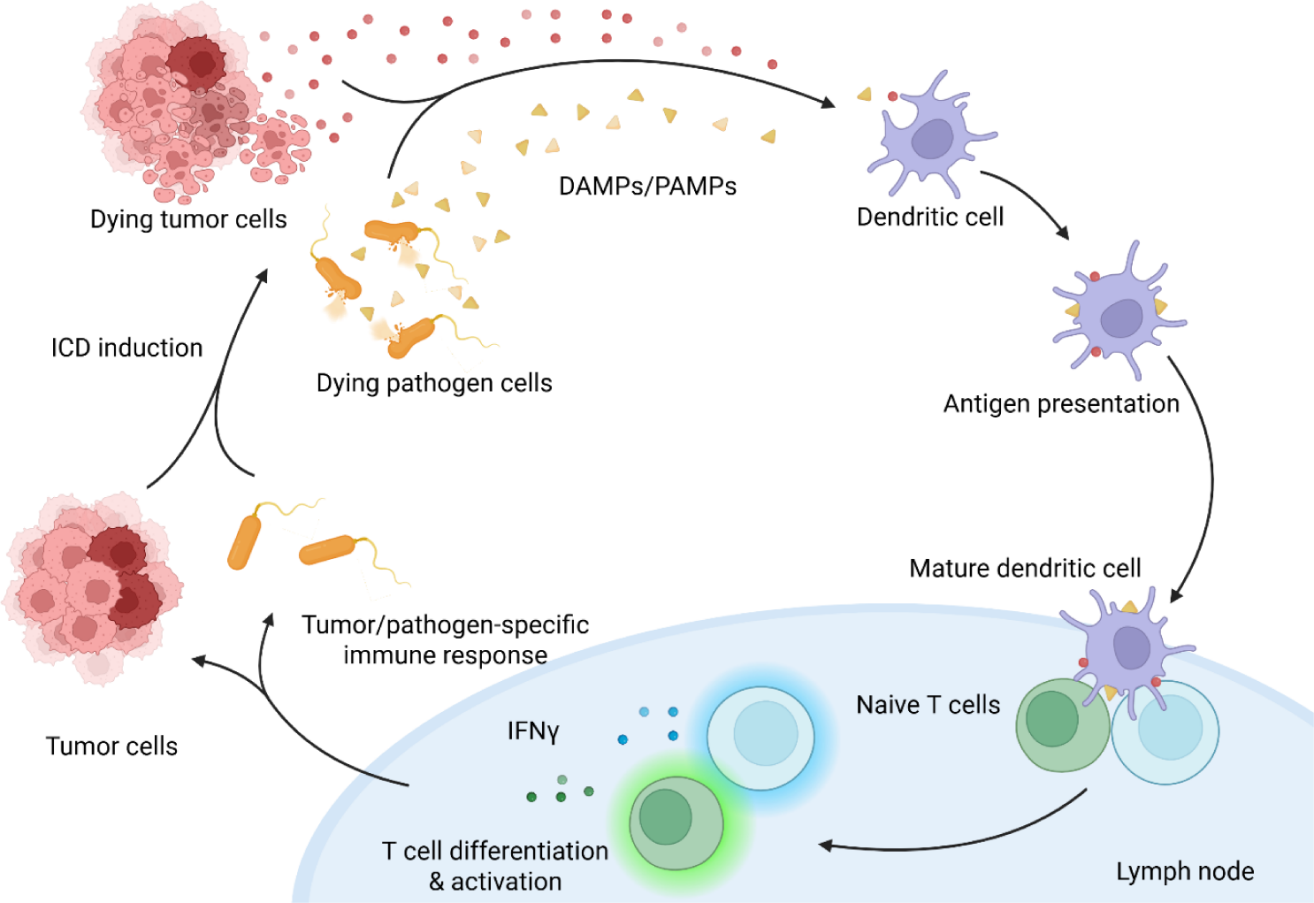
How immune stimulants (DAMPs/PAMPs) can bolster the immune response from peptide vaccinations. Following the death of a cancer cell, immune stimulants such as DAMPs and PAMPs are released and can bind to pattern recognition receptors (PRRs). The binding of PRRs enables DAMPs/PAMPs to interact with dendritic cells, leading to antigen presentation and the activation of CD4+ and CD8+ T cells. The activated T cells are then able to target additional cancer cells, leading to the death of these cells and initiating the cycle again.

Van Lint et al. explored the effectiveness of using TLR ligands within an mRNA vaccine to generate antitumor immunity and successfully showed that combining the TLR ligand with the antigen was able to slow tumor progression.^120^ While this vaccination strategy used mRNA, as opposed to peptides, a similar strategy of pairing TLR ligands with peptide antigens would likely lead to increased immune stimulation compared to delivering the antigen alone within the vaccine. However, additional studies are needed to determine how the TLR ligand and peptide antigen interact and to identify the optimal delivery mechanism to ensure that the two particles work together to stimulate the immune system.

Another method to aid in immune stimulation is to target DC subsets, allowing the antigen to have easier access to the DC. With the antigen having easier access to the DC, this should allow enhanced antigen presentation and increase the immune response to the vaccine. One strategy that has been explored is the use of ligands specific to DCs to target DC receptors.^121^ Another option for more targeted delivery of antigens to DCs is the use of chemokine receptors. A significant number of chemokine receptors are used to attract cells within the immune system. For example, the XRC1 receptor is a chemokine receptor that binds XCL1 to attract DCs to CTLs. By attracting DCs to CTLs, this increases the likelihood that the CTL will target and destroy the cell expressing the antigen of interest, as DCs have a higher chance of presenting the antigen to the CTL. Utilizing XRC1+ has been shown to increase the efficiency of antigen cross-presentation, and the inclusion of the XRC1 receptor has been shown to increase antitumor immunity in OVA-expressing tumor models.^122^ While this study did not consider the response of CD4+ T-cells, further research is needed to determine the true effectiveness of this strategy. An example of success utilizing DCs was shown by Carreno et al., who used DCs loaded with neoantigens that paired with low-frequency preexisting responses in melanoma patients.^123^ The neoantigen-specific responses within the patients were limited to the individual epitopes, but the TCR diversity against the antigens increased, suggesting that new clonotypes could be adequately primed.^123^ Despite the TCR diversity shown, no additional evaluations of this vaccine were conducted to explore whether these formulations could induce tumor regression.

Researchers have also explored the combination of multiple conjugates with different effectors or targeting motifs to boost multiple aspects of the immune response. For example, a TLR agonist can aid in DC maturation and activation, while a cell-penetrating peptide (CPP) can facilitate the antigenic domain’s access to the cytosolic compartments of DCs, where cross-presentation occurs. The combination of these two strategies can lead to an increase in the production of antigen-specific CD8+ T cells and enhanced antitumor immunity. An example of the value of combination therapies is found in recent work from Kimmel et al. on the design and evaluation of albumin-hitchhiking nanobody-STING agonist conjugates, which were shown to accumulate in tumors after systemic dosing and potentiate robust antitumor responses via activation of innate immunity, which generated strong adaptive immune cell proliferation and retention in tumor sites.^112^ Further, when combined with immune checkpoint blockade and adoptive cell therapy, this approach validated the impact that this technology has across different cancer subtypes, showing that innate agonist delivery platforms can prime both the tumor and splenic microenvironments for immune cell activation – offering a distinct advantage for evaluating this effect with the use of peptide-based vaccines and other adaptive immunotherapies. This strategy was investigated using HPV therapeutic mouse tumor models, and the researchers observed an increase in survival time and antigen-specific CD8+ T-cell infiltration within the tumor, along with a decrease in tumor size.^124^ This strategy has also been shown to break self-tolerance in nonhuman primates. Yet, we note that CPPs are nonspecific and can penetrate most cells.^125^ This could create unintended side effects as well as a decrease in bioavailability due to the adsorption of the drug by nonprofessional APCs.

Another strategy for success that is being explored for cancer vaccinations is to pair these therapeutic agents with chemotherapy or monoclonal antibody treatments. Cancer vaccinations can provide immune stimulation, while chemotherapy drugs can destroy cancer cells within the body. For example, Gall et al. detailed a clinical study in which patients with HER2-positive breast cancer were treated with either trastuzumab, a monoclonal antibody treatment, or a combination of trastuzumab and HER2-derived peptide vaccinations.^126^ The ongoing clinical trial has demonstrated positive results, with no recurrence in patients who received treatment with both trastuzumab and the cancer vaccination for up to approximately 34 weeks.^126^ Additional studies and the completion of this study are necessary to provide conclusive evidence, where preliminary results suggest that this strategy may be a promising method for reducing recurrence in patients. Another example of combining cancer treatment with drugs and cancer vaccination is the treatment of patients with gastric cancer with both chemotherapy and cancer vaccination.^127^ The study demonstrated longer survival times and slower tumor progression in patients who received cancer vaccination in combination with chemotherapy.^127^ However, the mechanism of chemotherapy can complicate the ability of combined immunotherapy. Some chemotherapies can cause immunosuppression as a side effect, leading to a decreased effectiveness of immunotherapy, while others can lead to the release of tumor antigens following the death of the cancer cells.^128^ Additional evaluations are needed to determine the best combination of immunotherapy with chemotherapy or monoclonal antibody treatment.

### Roadblocks to Peptide Cancer Vaccinations

Most of this review has focused on the many advances and advantages of peptide cancer vaccinations. However, we also need to quantify the challenges to peptide vaccination success that are the main reasons there have been no FDA approved peptide cancer vaccinations. There are three main mechanisms that have contributed to inefficient immune stimulation from peptide immunotherapy treatments – the immunosuppressive tumor microenvironment, immune evasion techniques within cancers, and tumor heterogeneity. All three of the mechanisms are inter-related and work together to assist tumor cells from avoiding immune surveillance and destruction. The general concepts for each of the mechanisms are illustrated in Figure 7.^129–133^

**Figure 7:**
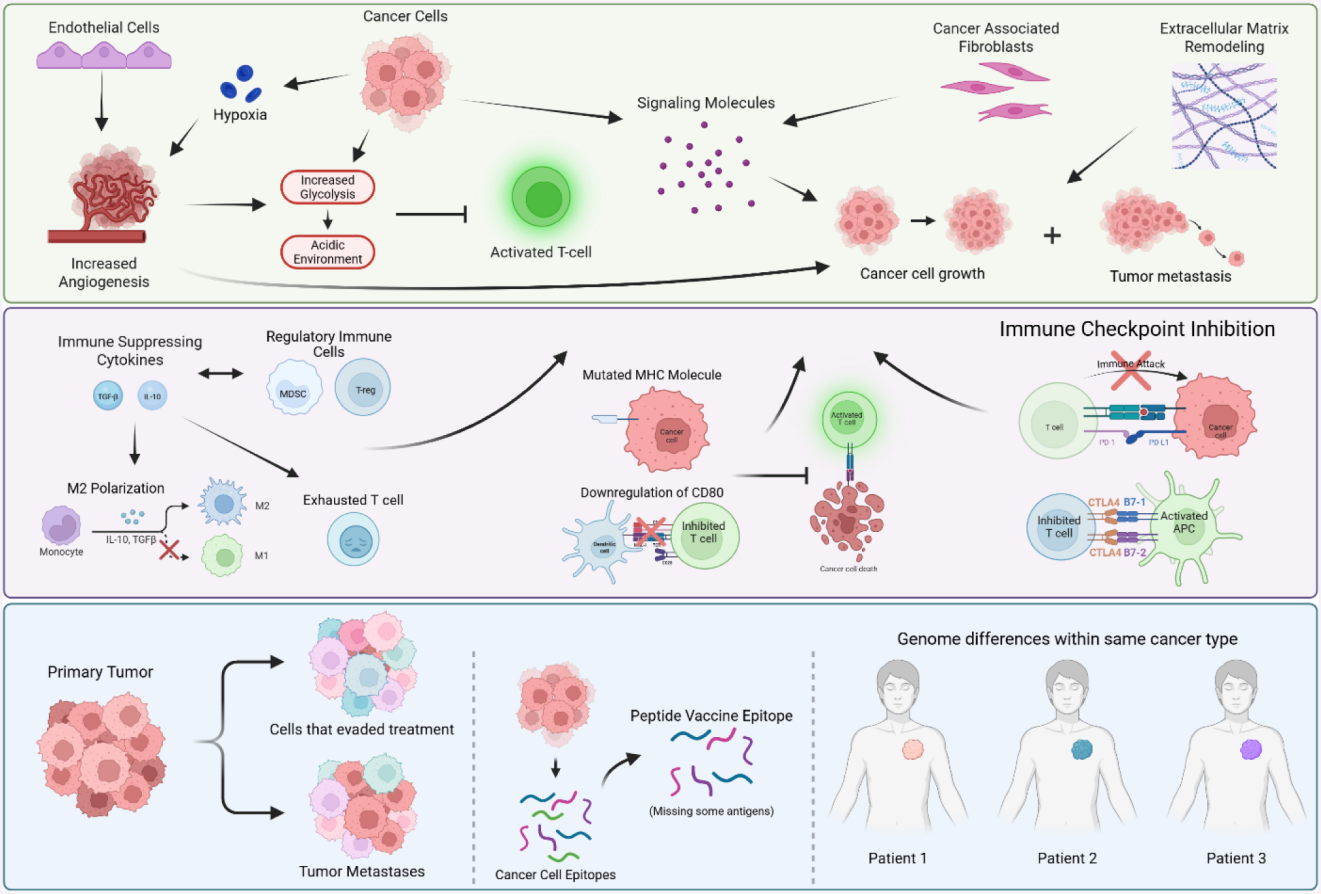
The different barriers that contribute to no peptide cancer vaccinations being FDA approved. TME is inter-related with immune suppression and evasion, and both work together to prevent tumor cells from being recognized and destroyed by the immune system. Tumor heterogeneity complicates the ability of cancer treatments to properly target and destroy all cancer cells, as the rapid mutations present can cause cancer cells to escape surveillance, leading to more proliferation and growth.

The tumor microenvironment (TME) is created by a combination of different cells and signaling molecules that work together to create immunosuppressive conditions that assist cancers in evading immune targeting. One characteristic within the TME is hypoxia, which is caused by cancer cells being unable to receive the necessary oxygen amount from blood vessels.^134^ Hypoxia leads to more aggressive metastasis of tumor cells due to the suppression of apoptosis, and the enhancement of angiogenesis, or the creation of new blood vessels.^134^ However, the TME creates more than just an environment that supports tumor growth. Within the TME, there is recruitment and polarization of immune cells that increases the amount of immunosuppressive phenotypes that present.^130^ Lymphocytes, macrophages, myeloid derived dendritic cells, and others are all examples of immune cells that are recruited to be within the TME.^133^ Macrophages provide a clear example of the mechanism that tumor cells use to hijack the immune system into being immunosuppressive. Tumor associated macrophages (TAMs) are many of the immune invading cells into tumors.^135^ However, tumors have taken advantage of invading macrophages to not only induce macrophage infiltration, but to create a bias for M2 polarization of the invading macrophages.^135^ M1 macrophages are pro-inflammatory macrophages, while M2 macrophages are immunosuppressive, assisting with tasks such as tissue repair and angiogenesis, both of which assist tumor development.^135^ TAMs are one distinct example of TME manipulating the immune system to support tumor growth as opposed to tumor regression.

The TME is also closely related to the different immune evasion mechanisms that are present within cancers. There is a wide range of different strategies that are utilized my tumor cells to avoid immune surveillance including secretion of immunosuppressing signaling molecules, recruiting regulatory immune cells such as Tregs, and expressing immune checkpoint molecules.^130^ The different immunosuppression mechanisms all contribute to the why behind a lack of robust immune responses to peptide cancer vaccinations. Even if the vaccine is able to lead to the presentation of the necessary antigens to T cells, the immunosuppressive TME means that those T cells will be unable to infiltrate and destroy tumor cells. Another key problem that has been identified within immune evasion is with T cell exhaustion. T cell exhaustion occurs from persistent T cell activation and causes problems such as T cells losing their effector functions, a decline in proliferation ability, and increased expression of inhibitory receptors.^136^ While significant advances have been made in understanding the biological processes that contribute to T cell exhaustion, there is still a lot more research that needs to be done. A recent study that contributed to a deeper understanding of how to combat T cell exhaustion found that the T cells with low-avidity contributes more to tumor immunity than high-avidity, essentially meaning that T cells that might be “less eager” to be activated are also less likely to become exhausted in the long run.^137^ The problem of the immune evasion is why combination therapies are the most likely route to FDA peptide vaccination approval. An immune checkpoint inhibitor, or other type of targeting therapy, can decrease the immunosuppressive environment while the peptide vaccination is able to train and prime the immune system to attack the cancer cells.

One final challenge that is present within peptide vaccinations is the problem of tumor heterogeneity. The differences within tumor genetics spans not only different cancer patients, but tumors within one cancer patient, complicating immunotherapy treatments that rely on specific markers or genes to be present within the tumor. For example, within nonsmall cell lung cancer, genomic studies have shown that there are genetic mutations present within brain metastases that are not present in the primary tumor.^138^ The issue of tumor heterogeneity is a current focus of cancer immunotherapy research, with a spotlight being on the development of single-cell multiomics, because it affects almost all immunotherapy treatments that are available.^139^ However, despite technological advances allowing for increased ability to predict biomarkers and patient responses to immunotherapy treatments, multiple barriers are in the way including cost, challenges in single cell isolation, and computational complexity of molecule profiling.^139^ The challenge of tumor heterogeneity is likely to be the most difficult to overcome. However, with increased computational power and strategies such as AI and machine learning, we believe that there will be multiple tools created that can provide valuable predictions to assist peptide vaccinations in covering a wider range of tumor epitopes. In the meantime, peptide vaccinations can adapt to tumor heterogeneity by incorporating a wider range of tumor epitopes within the delivered drug load and including combination therapies to assist in preventing tumor cells from escaping and mutating further.

## Conclusions

As research advances, the landscape of cancer treatment will become increasingly focused on personalized therapeutic methods that can train the immune system to recognize an individual’s neo-antigens and destroy cancer cells. For cancer vaccination to be successful, whether with or without peptides, researchers are encouraged to consider ways to activate both CD4+ and CD8+ T cells within the immune system and maintain the immune response long enough to allow immune cells to destroy the target cancer cells effectively. Many of the experiments discussed in this review were unable to achieve both, necessitating improvements to the vaccination method to engineer promising cancer treatments. In addition, the antigens used also determines the ability of immune cells to target the correct area. Neo-antigens are the most promising candidates for targeted delivery, as these peptide antigen fragments are unique to the cancer itself, reducing off-target delivery and effects. Being able to both identify unique neo-antigens and determine whether the antigens can generate a substantial immune response has proven to be difficult.

While in the past the focus of cancer vaccinations has been on whole-cell vaccines, the field has slowly transitioned into smaller drug loads including both mRNA and peptides. Following the COVID-19 pandemic, there was a sharp spike in research into mRNA vaccinations due to the emergency approval of two different mRNA vaccines. Peptide vaccinations have been behind mRNA research within popularity, however, there has been a consistent effort in developing new and innovative ways to use peptides to stimulate the immune system against cancer. Currently within the field, the two main focuses of research are on neo-antigen detection and identification, and on how to combat the immunosuppressive nature of tumors. Should these issues be solved, we believe that peptide vaccinations will quickly grow in popularity due to ease in which they can be manufactured, the longer circulation time of peptides within the body compared to mRNA, and the ability for peptides to be combined or conjugated with other drugs or therapies. Research into cell-penetrating peptides can also provide solutions for aiding peptides to enter cells, while solubility-enhancing technologies can help address the problems associated with insoluble peptides. The versatility of peptides also increases the likelihood of eventually creating personalized cancer vaccinations, which could enhance the survival rate for various cancer types, including those that receive limited research funding due to rarity in human populations. Increased screening and identification of different patient biomarkers could also contribute to the development of personalized medicines. Further research into biomarkers would also allow clinicians to predict how patients might respond to different treatments, including immunotherapy treatments.

While this review focuses on the applications of peptide vaccinations for cancer treatment, a wide range of other treatments could also be adapted for use with peptide vaccinations. Already, there has been research in peptide vaccinations to treat HIV,^98^ the field is expanding to explore utilizing peptide vaccines to treat influenza, malaria, and Hepatitis B.^140^ Adaptations in peptide delivery could provide the opportunity to provide cheaper options for vaccinations, especially in areas that struggle to afford the cost of what is considered standard care in developed nations. As more research into the development of peptide therapeutics is conducted, peptides have the potential to transform the current landscape of targeted drug treatments. Additionally, the continued integration of neoantigen discovery pipelines with synthetic biology platforms for antibody engineering for delivery in different parts of the body,^141^ bioconjugation-driven prodrug design,^142–144^ and innate agonist nanocarriers^117^ will be essential for developing next-generation peptide vaccines that can be readily tailored to maximize the impact for individual patients, while prioritizing the safety and potency of the formulation.
